# Hybrids of Nucleic Acids and Carbon Nanotubes for Nanobiotechnology

**DOI:** 10.3390/nano5010321

**Published:** 2015-03-12

**Authors:** Kazuo Umemura

**Affiliations:** Biophysics Section, Department of Physics, Tokyo University of Science, 1-3 Kagurazaka, Shinjuku, Tokyo 1628601, Japan; E-Mail: meicun2006@163.com; Tel.: +81-352288228

**Keywords:** single-stranded DNA, double-stranded DNA, RNA, carbon nanotubes (CNTs), functionalization

## Abstract

Recent progress in the combination of nucleic acids and carbon nanotubes (CNTs) has been briefly reviewed here. Since discovering the hybridization phenomenon of DNA molecules and CNTs in 2003, a large amount of fundamental and applied research has been carried out. Among thousands of papers published since 2003, approximately 240 papers focused on biological applications were selected and categorized based on the types of nucleic acids used, but not the types of CNTs. This survey revealed that the hybridization phenomenon is strongly affected by various factors, such as DNA sequences, and for this reason, fundamental studies on the hybridization phenomenon are important. Additionally, many research groups have proposed numerous practical applications, such as nanobiosensors. The goal of this review is to provide perspective on biological applications using hybrids of nucleic acids and CNTs.

## 1. Introduction

**Figure 1:**
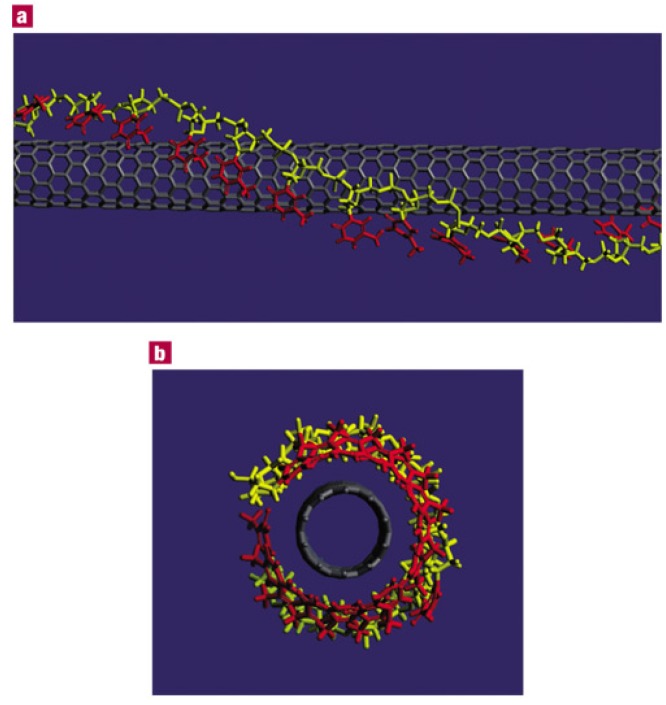
A theoretical model of structure of single-stranded DNA (ssDNA) and single-walled carbon nanotubes (SWNTs) hybrids, proposed by Zheng *et al.* (10,0) SWNT and poly(T) were assumed for the calculation (Reprinted from reference [1] with permission).

The hybridization of DNA and carbon nanotubes (CNTs) was first reported in 2003 when Zheng *et al.* presented the hybridization of single-stranded DNA (ssDNA) and single-walled carbon nanotubes (SWNTs) [1]. They not only presented experimental data, but also introduced a theoretical model of the adsorption of DNA onto SWNT surfaces (Figure 1). Furthermore, this work suggested there is a correlation between specific bases of DNA molecules and SWNT chirality. Nakashima *et al.* independently described the solubility of SWNTs with DNA molecules in 2003 [2] using double-stranded DNA (dsDNA) molecules. Thereafter, many research groups have reported on the hybridization of DNA and CNTs, especially SWNTs.

There have been two central hybridization research goals since 2003. The first is to improve the solubility of CNTs. Although the unique structural and physicochemical properties of CNTs are appealing for fundamental and industrial applications, uncoated CNTs typically form insoluble bundles in aqueous solutions. To take full advantage of CNTs, they must be prepared in a mono-dispersed form, which is especially true for SWNTs. Mono-dispersed CNTs can be prepared with attached hydrophilic organic molecules, such as sodium dodecyl sulfate (SDS), peptides, proteins, or DNA molecules, which can enhance CNT solubility. Another goal of hybridization is to purify SWNTs. SWNTs exhibit chirality, which is determined by the chirality vector, *C_h_* = *na*_1_ + *ma*_2_. The *a*_1_ and *a*_2_ variables represent unit vectors on a graphene surface, and *n* and *m* are integers. Physical properties of SWNTs, such as electrical and optical characteristics, can vary depending on chirality. Because the chirality of SWNTs cannot be well controlled during synthesis, post-synthesis purification is required for some SWNTs for industrial applications. These two objectives were the main rationales for initial studies on hybridization.

While the method for DNA adsorption onto CNTs was originally developed to solubilize and purify CNTs, DNA-CNT hybrids also have great potential as DNA nanodevices. In this review, the fundamental studies of DNA and CNT hybridization for biological applications, especially in solutions, will be presented. Modification of electrode surfaces with DNA and CNTs is another major area of research; however, this topic will be explored elsewhere.

## 2. Non-Covalent Hybridization of DNA Molecules onto SWNT Surfaces

The solubility of CNTs is a major area of CNT research, with the aim of preparing mono-dispersed CNT suspensions. Prior to using DNA, various organic molecules were employed to hybridize with CNTs [3,4,5,6,7,8,9,10,11,12,13,14,15,16,17,18]. Although this article focuses on DNA technology, several examples of hybridization using other molecules are introduced for comparison. Various organic molecules, such as polyvinyl pyrrolidone (PVP) and polystyrene sulfonate (PSS) [3], poly(propionylethylenimine-co-ethylenimine) [5], hyperbranched polymers [6], 12-membered cyclodextrins [7], SDS [8], Nafion [10], poly(*N*-cetyl-4-vinylpyridinium bromide-co-*N*-ethyl-4-vinylpyridinium bromideco-4-vinylpyridine) [14], and electroactive methylene blue (MB) dyes [15] have been employed. Polysaccharides, such as lactose-appended schizophyllan [12] and *N*-trimethyl-chitosan [17], have also been used to control the electrical properties of prepared hybrids. Furthermore, proteins such as bovine serum albumin (BSA) [4] have been employed to solubilize both SWNTs and multiple-walled CNTs (MWNTs). Early reports suggested the possibility of separating specific CNTs using surface modification.

Numerous review articles have been published on the subject of solubilization [19,20,21,22,23,24,25,26,27,28,29,30,31,32,33,34,35,36,37,38]. At present, the review titled “*Advances toward Bioapplications of Carbon Nanotubes*” and prepared by Lin *et al.* in 2004 has been cited over 500 times [24]. Although this review described examples of solubilization studies using various organic molecules, only a few studies illustrating the relationship between DNA and CNTs existed before 2004 because the review was written at the early stages of research on DNA and CNT hybridization. Since 2005, several review articles that have focused on DNA applications with CNTs have been published [25,27,29,30,31,32,34,35,36,38].

In 2014, The Web of Science database contained approximately 4000 studies with both the keywords “DNA” and “carbon nanotube”. Among these, approximately 1000 were reports on suspensions or solutions. In this review, approximately 240 papers were selected that focus on biological applications. First, the fundamental studies of hybridization are reviewed [39,40,41,42,43,44,45,46,47,48,49,50,51,52,53,54,55,56,57,58,59,60,61,62,63,64,65,66,67,68,69,70,71,72,73,74,75,76,77,78,79]. The articles are divided into categories based solely on the types of nucleic acid molecules used.

Interestingly, although some of the articles provided detailed information on the DNA molecules that were used in the experiments, some of them did not include details on the DNA molecules. If the types of the employed DNA molecules were important factors for the research, the authors would likely have described them in detail. However, if the DNA molecules were merely one of the organic molecules used to solubilize CNTs, detailed descriptions might not be necessary. A researcher’s stance on DNA-CNT hybrids is apparent based on the description of DNA in an article.

## 3. Hybridization with dsDNA

A large portion of hybridization research uses double-stranded DNA molecules, including DNA from salmon testes [2,39,54,67,70,72], calf thymus [74,76,79], or fish sperm [75]. These commercially available DNA molecules are suitable for industrial applications because their cost is less than that of synthesized ones. At the initial stages of hybridization research, finding optimal hybridization conditions was one of the major aims. Barisci *et al.* proposed that a 1% SWNT suspension could be prepared with DNA molecules [39]. Characterization of the prepared DNA-CNT hybrids was the next area of study. Badaire *et al.* measured the average length and diameter of the hybrids using dynamic light scattering [40]. Cathcart *et al.* reported an increase in the photoluminescence intensity of SWNTs with decreasing concentrations of SWNTs [54].

Several applications for DNA-CNT hybrids have been reported. Asada *et al.* fabricated thin-film transistors (TFTs) using dsDNA-SWNT hybrids [67]. Wang *et al.* prepared transparent conductive films on PET substrates [70]. Wang *et al.* modified a glassy carbon electrode with dsDNA-SWNT hybrids and Nafion composites [75].

Although the hybridization method is essentially well established, the search for optimal hybridization conditions is ongoing. Chhikara *et al.* established that cationic cholesterol induces higher stability in DNA-SWNT hybrids [74]. Ao *et al.* reported cholesteric and nematic liquid crystalline phase behavior of SWNTs with dsDNA [72]. Primo *et al.* dispersed bamboo-like multi-walled carbon nanotubes (bCNTs) for the modification of glassy carbon electrodes [76]. Recently, Tardani *et al.* demonstrated the hybridization in a nematic phase of dsDNA-water-NaCl [79]. These reports suggest that optimal conditions of the hybridization are easily changed according to types of DNA/CNT and concentrations of DNA/CNT mixtures.

As described above, various fundamental and applied studies have been reported using natural dsDNA molecules. Variation of dsDNA molecules revealed huge potential for industrial applications. However, the mechanisms of hybridization between dsDNA and CNTs are currently not well understood. Fundamental research clarifying the details of dsDNA-CNT hybrids is necessary. Furthermore, many studies do not identify the sequences of dsDNA molecules used. Examining the effects of the sequence of dsDNA used could be a focus of future study.

## 4. Hybridization with ssDNA

Hybridization of ssDNA and CNTs has also been demonstrated. In most cases, synthesized oligonucleotides were utilized. Because Zheng *et al.* reported that the 30-m of thymine (T_30_) resulted in the highest yield of hybridization, oligonucleotides containing thymine have been widely employed.

Hu *et al.* used (GT)_15_ and achieved selective and sensitive detection in the form of quasi-reversible voltammetric responses [43]. Bertoncini *et al.* employed a poly (GT)_15_ sequence to confirm the debundling of SWNTs [53]. Haggenmueller *et al.* compared (GT)_15_, (GT)_10_, (AC)_15_, (AC)_10_, and C_10–30_ as well as surfactants and other biomolecules in hybridization [61]. Hughes *et al.* used A_15_, G_15_, C_15_, and T_15_ to disperse CNTs and found that the order of dispersion efficiency was T > C > G >> A, and that the adsorption speed of cytosine was the fastest [68].

The use of synthesized ssDNA molecules enabled detailed characterization of the physical and chemical properties of the prepared hybrids. Fagan *et al.* used the 30-m 5'-GT(GT)13GT-3' to investigate the effects of pH on hybrid properties [46]. Jin *et al.* used (GT)_15_, (GC)_15_, and 5'-TAG CTA TGG AAT TCC TCG TAG GCA-3' to detect the transition phenomenon between 0 and 50 °C [58]. Cooper *et al.* debundled double-walled CNTs (DWNTs) using ssDNA and evaluated the DWNTs using cyclic voltammograms [63]. Ghosh *et al.* utilized ssDNA, such as (GT)_40_, to disperse MWNTs while studying thermal ablation [64]. Jovanovic *et al.* used gamma-irradiation for hybridization instead of sonication [65]. Qiu *et al.* used (GT)_20_ and investigated electrostatically driven interactions using force-distance curves [77].

Throughout, technical progress has been reported. Chen *et al.* demonstrated controlled precipitation using T_30_ [45]. Onoa *et al.* fabricated a field effect transistor (FET) with poly(CT) and SWNTs [51]. Zhao *et al.* hybridized SWNTs with long ssDNA molecules synthesized by rolling circle amplification [52]. Han *et al.* used (GT)_29_SH for hybridization with SWNTs and attached Au nanoparticles to the prepared hybrids [55]. Hopkins *et al.* demonstrated the alignment of ssDNA-SWNT hybrids using an inkjet printing method [56]. Pease *et al.* investigated the length distribution of SWNTs using electrospray differential mobility analysis (ES-DMA).

Short ssDNA molecules with user-specified lengths and sequences can be easily synthesized. Therefore, comparative studies using multiple combinations have been reported for understanding the hybridization mechanism. In addition, examination of assorted combinations can be used to obtain clues for sorting and purifying CNTs. The focus of many of the studies using synthesized ssDNA molecules was closer to chemistry than to biology. Further work from the viewpoint of biology and biotechnology are anticipated in the near future.

## 5. Comparative Studies Using ssDNA, dsDNA, and Other Nucleic Acids

Investigations comparing ssDNA, dsDNA, and other nucleic acids are also popular approaches to studying hybridization. He *et al.* deposited calf thymus dsDNA, calf thymus ssDNA, poly(G), or poly(diallyldimethylammonium) (PDDA) on water-soluble oxidized SWNTs to generate DNA sensors [41]. Yan *et al.* compared the hybridization of MWNTs with either calf thymus ssDNA or dsDNA using cyclic voltammetry [44]. Gigliotti *et al.* found that long genomic ssDNA molecules formed tight helices around CNTs [47]. Gladchenko discussed the behaviors of double-stranded and single-stranded regions of ultrasonically examined dsDNA present in the hybrid [48]. Vogel *et al.* examined (GT)*_n_* and (AC)*_n_* with *n* = 2, 3, 5, 10, 20, or 40 to find the optimum conditions for hybridization [59]. Yamamoto *et al.* used short dsDNA molecules, A_20_/T_20_, as well as A_20_ and T_20_ to purify highly stable hybrids by chromatography [71].

RNA molecules were examined by several researchers. Ishibashi *et al.* employed RNA molecules, poly(A), poly(C), and poly(G) to prepare RNA-SWNT hybrids, and fabricated multilayer films using the layer-by-layer technique [50]. Sanz *et al.* hybridized RNA molecules from baker’s yeast with SWNTs, DWNTs, and MWNTs for use in gene delivery systems [73].

There are unique approaches using specific molecules that are related to DNA or RNA. Ikeda *et al.* prepared SWNT hybrids with adenosine triphosphate (ATP) and other related molecules using a high-speed vibration milling technique [49]. Schmucker modified DNA molecules with pyrene and prepared DNA-SWNT hybrids in 2013, revealing that DNA modification affected the solubilization and photoluminescence of SWNTs [78].

Comparative studies using both ssDNA and dsDNA molecules are attractive to illuminate the differences of the nucleic acid structures on CNT surfaces. When naturally derived long dsDNA molecules are employed, preparation of ssDNA molecules that have similar length and similar sequence is not easy. When dsDNA molecules are synthesized using hybridization of two short synthesized ssDNA molecules, residual ssDNA molecules should be considered. The use of well-purified ssDNA and dsDNA molecules that are 500 to 1000 bp long and have similar sequences could be an interesting area of research. There are not many examples of hybridization of RNA and other nucleic acids with CNTs. The use of various nucleic acids with diverse physical properties is an attractive target in biophysics.

## 6. Selective Adsorption and Separation of CNTs

Zheng *et al.* described the selective adsorption of DNA molecules that was dependent on the type of DNA base, and they indicated the possibility of separating SWNTs by attaching DNA molecules [1]. This idea has been followed up in numerous recent papers. When a study uses more than one ssDNA sequence for hybridization with SWNTs, selective adhesion is expected [41,44,47,50,58,59,67,71]. Albertorio *et al.* specifically focused on selective adhesion, and they found that the binding strengths between nucleobases and SWNTs followed the order G > C > A > T [80].

Multiple studies have reported on the separation of specific SWNTs using selective adhesion of DNA molecules [50,59,61,68,80,81,82,83]. Currently, the chirality of SWNTs cannot be well controlled during synthesis. A technique for separating SWNTs is one of the major aims of CNT research [62,84,85,86,87,88,89,90,91,92,93]. For example, Vetcher *et al.* separated DNA/RNA-SWNT hybrids using agarose gel electrophoresis [84].

**Figure 2:**
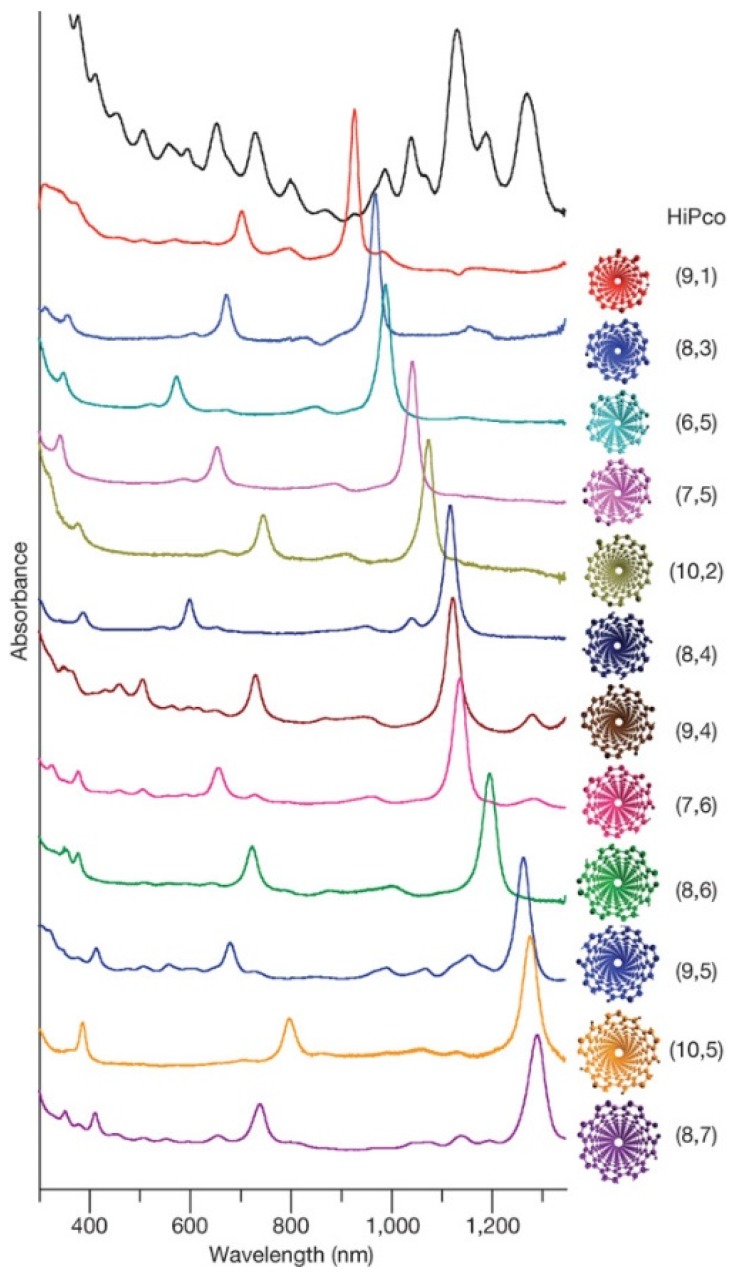
Optical absorption spectra of separated SWNTs by selective adsorption of ssDNA, reported by Tu *et al.* (Reprinted from reference [91] with permission).

Chromatography techniques have also been used to purify or separate DNA-CNT hybrids. Bauer *et al.* prepared three types of hybrids: octadecylamine functionalization (SWNT-ODA) in tetrahydrofuran (THF), butyl group functionalization (SWNT-butyl) in THF, and DNA-SWNT hybrids. Then, the hybrids were separated by size-exclusion chromatography (SEC) in 2007 [85]. They also found that the molar mass of the hybrids was proportional to the rod length of the SWNTs [87]. Chun *et al.* used flow-field flow fractionation (flow-FFF) to separate DNA-SWNT hybrids [88]. Asada *et al.* reported the separation of DNA-SWNTs by length and the separation of DNA-DWNTs using size-exclusion high-performance liquid chromatography (HPLC) [90]. Much progress using chromatographic methods for separation was demonstrated by Tu *et al.* in 2009 [91] where they examined many combinations of ssDNA molecules with specific sequences and SWNTs with various chiralities. They found that 12 major single-chirality semiconducting SWNTs could be separated by ion-exchange chromatography, and the results of the separation are clearly indicated in the absorption spectra (Figure 2).

Although most separation studies focus on hybrids of SWNTs, Kim *et al.* reported DWNT hybrids as a function of nanotube diameter, suggesting that stronger van der Waals forces between large-diameter tubes were an important factor [89]. Shakhmaeva *et al.* proposed a unique method of removing DNA molecules from CNT surfaces. Using divalent metal ions, the state of plasmid DNA changed from supercoiled to relaxed form and regulated the adhesion of DNA molecules onto the CNTs [92]. Tardani *et al.* presented the effects of polymers and ionic strength on the phase separation of DNA-SWNT suspensions [93].

Separation and purification studies have been an area of intense focus because they are absolutely vital for progress in CNT nanotechnology. If separation techniques can be applied to DNA nanotechnology, this may open a new field of study.

## 7. Characterization of DNA-CNT Hybrids

The structural, physical, and chemical properties of DNA-CNT hybrids have been characterized by various methods, including UV-Vis spectroscopy (UV-Vis), near infrared (NIR) spectroscopy, Raman spectroscopy, photoluminescence (PL), transmission electron microscopy (TEM), scanning electron microscopy (SEM), scanning probe microscopy (SPM), and agarose gel electrophoresis. Because SWNTs have unique optical properties, spectroscopic studies are a prevalent approach to study hybrids [41,42,46,48,54,71,81,94,95,96,97,98,99,100,101,102,103,104,105,106,107,108,109,110,111,112,113,114,115].

Raman spectroscopy has been utilized since the establishment of hybridization research. Glamazda *et al.* described spectroscopic differences between DNA-SWNTs and SDS-SWNTs using Raman spectroscopy in 2006 [94]. Shoda *et al.* reported a decrease in the G-band in Raman spectra with DNA adsorption, only in the case of metallic tubes [106]. Simpson *et al.* performed Raman spectroscopy of length-separated fractions of ssDNA-SWNT hybrids to evaluate the role of defects in the fluorescence quantum yield of SWNTs [107]. Chen *et al.* attached silver nanoparticles onto DNA-SWNT hybrids to measure surface-enhanced Raman scattering (SERS). From the SERS measurements, they suggested that DNA-SWNT hybrids could be useful as nanoprobes for marking cells [108].

Jeng *et al.* suggested that an ssDNA-SWNT hybrid concentration of 6 nM could be detected using NIR band-gap fluorescence [95]. Asada *et al.* performed various types of spectroscopies involving NIR to assess charge transport on DNA-SWNT hybrids [98]. Karachevtsev *et al.* achieved glucose sensing by measuring the NIR spectra of DNA-SWNT hybrids using enzyme reactions [99]. Tu *et al.* reported a redox reaction of ssDNA-SWNT hybrids, suggesting that the NIR signals were tunable by the type and pH of the buffer solutions used [100]. Xu *et al.* described the potential of DNA-SWNT hybrids in immunoassays and glucose sensing as DNA-SWNT hybrids were optically sensitive to H_2_O_2_ and glucose in the NIR measurements [101]. Cao *et al.* suggested that red shifts in NIR absorption spectra were significant in hybrids with semiconducting SWNTs, but not in those with metallic SWNTs [102]. Noguchi *et al.* found that the optical properties of the dsDNA-SWNT hybrids could be modulated by changing the pH of the solution [81]. Ozturk *et al.* performed Raman spectroscopy on DNA-SWNT hybrids that were prepared with poly(A), poly(T), poly(G), and poly(C) at different lengths. Their results indicated that although the kinetics of DNA adsorption was affected by DNA length, the electronic properties of the DNA-SWNT hybrids were dependent on the species of DNA bases used [114]. Hobbie *et al.* examined the properties of G_30_ NIR [42], and Gladchenko *et al.* combined Raman spectroscopy with NIR to evaluate sonicated dsDNA that contained ssDNA regions [48]. Ishibashi *et al.* also used UV-Vis-NIR and Raman spectroscopy for comparative studies of poly(A), poly(C), and poly(G) [50].

**Figure 3:**
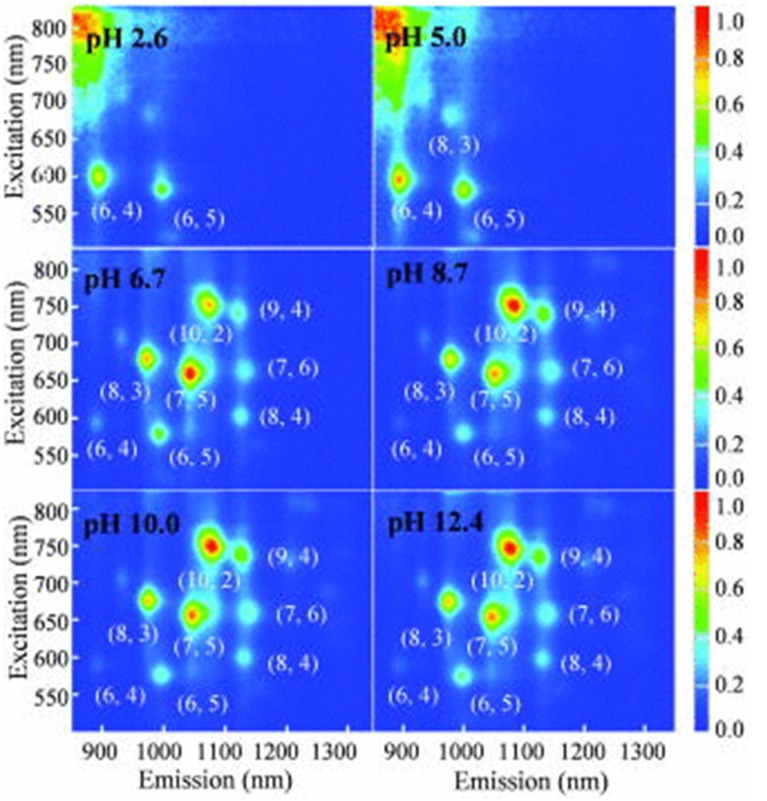
Differences in photoluminescence (PL) maps due to the pH values of solvents indicated by Kim *et al.* (Reprinted from reference [89] with permission).

Lacerda *et al.* demonstrated that nanotube aggregation and bundling led to decreased PL [97]. Cathcart *et al.* examined the aging effects of the DNA-SWNT hybrids on PL, observing that between 20 and 50 days after sample preparation, the PL intensity of the hybrids increased [103]. They also reported an increase in the PL intensity of SWNTs with decreasing concentrations of SWNTs, as mentioned in the previous section [54]. Schmucker discussed the PL spectra of hybrid DNA molecules modified with pyrene and SWNTs [78]. Ignatova *et al.* suggested fluorescence resonance energy transfer (FRET) could occur between two rare earth ions and the ionized phosphate groups of DNA [110]. Shao *et al.* combined a classical ruthenium (II) complex with guanine to yield tunable PL materials [111]. Hughes *et al.* used PL to compare 15-m of A, G, C, and T [68]. Figure 3 shows the changes in PL spectra due to solvent pH that were reported by Kim *et al.* [89]. Such phenomena are expected to apply to optical switches.

Other types of spectroscopic measurements were used by numerous investigators. Park *et al.* studied the kinetics of ssDNA adsorption onto SWNT surfaces using surface plasmon resonance imaging (SPRi) and demonstrated the effects of length and buffer using SPRi [109]. Badaire *et al.* used dynamic light scattering to estimate the length and diameter of the hybrids [40]. Tardani *et al.* used circular dichroism and rheology to study dsDNA-SWNT hybrids, as cited in the previous section [79]. He *et al.* systematically carried out various characterization methods, such as SEM, TEM, UV-Vis spectroscopy, Raman spectroscopy, and thermogravimetric analysis (TGA), to study positively and negatively charged hybrids [41].

Microscopic techniques are also powerful tools to directly characterize DNA-CNT hybrids [56,83,98,99,113,116,117,118,119,120,121,122,123,124,125,126]. Although TEM and SEM are major techniques, they are too broad to be adequately summarized in this section. A specific review article for using electron microscopy for studying DNA-CNT hybrids will be compiled. Here, several studies that used SPM and other specific microscopies as their main characterization methods are introduced.

Takahashi *et al.* published an article that specifically focused on atomic force microscopy (AFM) titled “*AFM Imaging of Wrapped Multiwall Carbon Nanotube in DNA*” in 2006. They examined dsDNA-MWNT hybrids using AFM on highly oriented pyrolytic graphite (HOPG) surfaces, and concluded that the diameters of the MWNTs strongly affected the adsorption of DNA [116]. Campbell *et al.* observed a regular pattern on the surface of CNT-DNA hybrids by AFM. Furthermore, they attached core/shell CdSe/ZnS quantum dots to the DNA-SWNTs using thiol-modified DNA molecules, and the attachment of the quantum dots was also confirmed by AFM [117]. Toita *et al.* prepared two types of SWNTs that were synthesized using high-pressure carbon monoxide (HiPco) and arc discharge (Arc) methods. Based on AFM measurements, they suggested that the adsorption mechanism of DNA molecules was affected by the diameter of SWNTs [118]. Hayashida *et al.* hybridized ssDNA or dsDNA using HiPco or chemical vapor deposition (CVD) methods and compared the thickness of the DNA-SWNT hybrids using cross-sections of AFM images. They found that the manner of adsorption was significantly different among the four combinations of hybridization [122]. Nii *et al.* studied the adsorption and desorption of dsDNA onto/away from SWNTs that were functionalized with polyethylene glycol (PEG), which was confirmed by cross-sectional analysis of AFM images and agarose gel electrophoresis [113]. Using a similar experimental approach, Nii *et al.* also reported the selective binding of ssDNA-binding (SSB) proteins to ssDNA-SWNTs, but not to dsDNA-SWNT hybrids [123].

**Figure 4:**
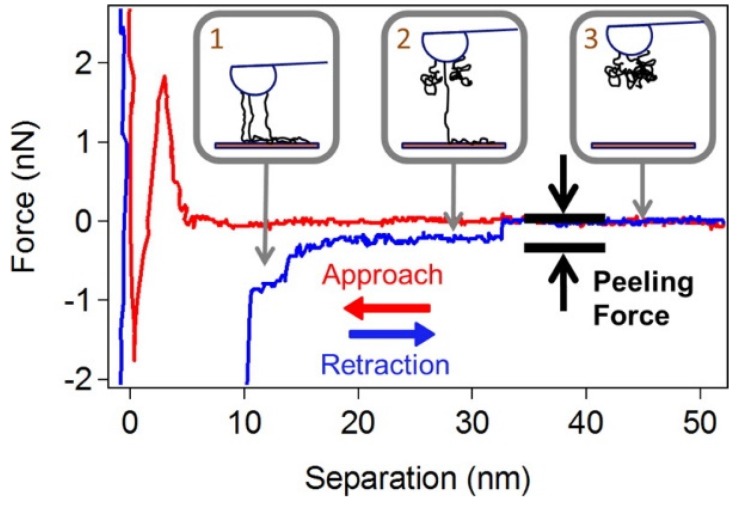
A typical force curve during the peeling of a T_100_ ssDNA molecule away from a SWNT surface, demonstrated by Iliafar *et al.* (Reprinted from reference [83] with permission).

Force spectroscopy is an advanced mode of AFM. Using force spectroscopy, Iliafar *et al.* found that the binding strength of ssDNA to curved SWNTs is much greater than that to flat graphite [83]. Figure 4 shows an example of their force spectroscopic data. Moreover, they estimated the free energy of DNA binding to SWNTs for every nucleic base. Qiu *et al.* carried out force spectroscopy to measure the electrostatic forces between GT_20_ and SWNTs [77].

Several unique approaches related to microscopic methods have been demonstrated. Xu *et al.* investigated the motion of SWNTs in solution using optical trapping techniques and scanning photocurrent microscopy [119]. For measurement, microbeads were attached to SWNTs via DNA molecules. The study demonstrated that the surface potential against Au substrate varied depending on the type of SWNTs. Arnett *et al.* reacted ssDNA ligase with DNA-CNT hybrids that were prepared with pyrimidine-functionalized CNTs and observed the formation of a 3D lattice-like CNT structure using wet-cell TEM [120]. Hayashida *et al.* performed Kelvin force microscopy to measure the surface potential of ssDNA-SWNT hybrids and other related samples [121].

**Figure 5:**
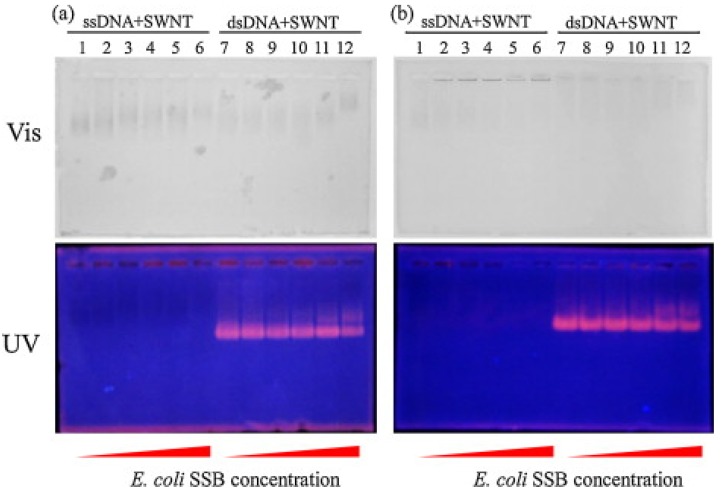
An example of agarose gel electrophoresis of DNA-SWNT hybrids with and without ssDNA-binding (SSB) proteins as performed by Nii *et al.* (Reprinted from reference [123] with permission).

Electrophoresis methods are popular in biology and have been applied to characterize DNA-CNT hybrids. For example, Vetcher *et al.* employed agarose gel electrophoresis to characterize separated DNA/RNA-SWNT hybrids [84]. Nii *et al.* combined staining of DNA molecules using ethidium bromide (EtBr), of proteins with Coomassie Brilliant Blue (CBB), and direct observation without staining for SWNTs to characterize DNA-SWNT hybrids after SSB reaction [123]. Figure 5 shows the results of the electrophoresis presented by Nii *et al.*, where significant differences between ssDNA and dsDNA were observed in the electrophoresis patterns.

Fluorescent spectroscopy and fluorescent microscopy revealed a peculiar quenching phenomenon [127,128,129,130,131,132,133,134,135,136,137]. Rao *et al.* confirmed the binding of poly(rU) molecules using single-molecule fluorescence microscopy and Raman spectroscopy [127]. Yang *et al.* described carbon nanotube-quenched fluorescent oligonucleotides and hairpin-structured fluorescent oligonucleotides [128]. Zhang *et al.* detected mercury ions (Hg^2+^) in an aqueous solution using fluorescence quenching [129]. Zhao *et al.* prepared a reusable fluorescent sensor using DNA-SWNT hybrids for highly sensitive and selective detection of Ag^+^ and cysteine (Cys) in aqueous solutions [130]. D’Souza *et al.* used ssDNA hybrids to measure electron transfer in self-assemblies of ion-paired porphyrins employing (6,5) and (7,6) semiconductive SWNTs [131]. Martinez *et al.* found that streptavidin was an effective linker between SWNTs and oligonucleotides, and therefore, streptavidin efficiently prevented the non-specific adsorption of high-affinity molecules, such as DNA, onto CNTs [134]. Judkins *et al.* performed EPI-fluorescence microscopy (EFM) on DNA-SWNT hybrids to estimate their diffusion constants [137]. In this study, SWNTs were non-covalently coated with 1-pyrenebutanoic succinimidyl ester (PSE) as a fluorophore, and then DNA molecules were covalently attached to the PSE. This study suggested these tools could be used in the development of drug delivery systems.

There has been much work done in characterizing the physical and chemical properties of DNA-CNT hybrids. This is especially true for various nanomaterials where specific optical properties of CNTs seem to depend on CNT chirality and DNA sequence. A future application of this work might include development of optical sensors and switches. However, it is important to note that when a fluorescent dye is linked to DNA, quenching can occur on the CNT surface. If there are excess unbound DNA molecules nearby, this quenching can be difficult to observe.

These studies also indicate the importance of single-molecule measurements. In order to determine specific physical properties of CNTs, further progress using single-molecule techniques is needed. For example, if single-molecule electrophoresis were possible for DNA-CNT hybrids, this would be a powerful method to characterize DNA-CNT hybrids.

## 8. Functionalization of CNTs for DNA Attachment

The use of functionalized CNTs for DNA attachment is a prevalent approach in hybridization research [138,139,140,141,142,143,144,145,146,147,148,149,150]. In most cases of this approach, DNA molecules are covalently attached onto the functionalized CNTs. CNT structures should be chemically modified, and therefore, the physical and chemical properties of the CNTs should also be different in comparison with those of bare CNTs.

Hazani *et al.* attached ssDNA molecules onto SWNT surfaces using carbodiimide-assisted amidation in 2003 [138]. They characterized the samples using UV-Vis spectroscopy and confocal fluorescence microscopy and revealed hybridization of the ssDNA-SWNT hybrids with complementary ssDNA. Stevens *et al.* prepared fluorinated SWNTs (fluoronanotubes) to covalently attach various molecules such as DNA in 2003 [139]. Singh *et al.* synthesized conjugates of peptide nucleic acid (PNA, an artificial analogue of DNA) and SWNTs [141]. Wang *et al.* functionalized SWNTs with carboxylic groups and DNA molecules with amino groups and hybridized them using amide linkages [142]. Yang *et al.* covalently attached DNA molecules onto oxidized SWNT surfaces in organic and aqueous solutions. The attachment of the DNA molecules was confirmed by TEM using complementary DNA molecules that were functionalized using gold nanoparticles with ssDNA-SWNT conjugates [144]. Moghaddam *et al.* formed photo-adducts on graphitic surfaces using *N*-5-azido-nitrobenzoyloxy succinimide (ANB-NOS) and covalently attached DNA molecules that were terminated with amine groups [145]. Alidori *et al.* prepared ammonium-functionalized CNTs for DNA attachment [146]. Dolash *et al.* proposed a model in which covalent bonding was formed between DNA and CNTs using sonication [147]. Singh *et al.* developed the covalent double functionalization method using oxidized SWNTs with a combination of purine-pyrimidine and purine-purine nucleobases [148]. Canete-Rosales *et al.* fabricated hybrids of single-stranded deoxyoligonucleotide oligomers (DNO) and CNTs using both covalent and non-covalent attachment [149]. Kaufmann *et al.* functionalized MWNTs with hydroxyl groups [150], subsequently attaching a carrier strand oligodeoxynucleotide (CS-ODN) to the functionalized MWNTs and hybridizing a therapeutic antisense oligodeoxynucleotide (AS-ODN) with the conjugates. Finally, Yuan *et al.* reviewed the various functionalization methods [143].

Although not a chemical functionalization method of CNTs, the insertion of DNA molecules into CNTs is a unique idea [36,69,124,126,151,152,153,154,155,156,157]. For example, Gao *et al.* predicted that a DNA could possibly be spontaneously inserted into CNTs using molecular dynamics (MD) simulations [151]. Okada *et al.* demonstrated insertion of ssDNA molecules into SWNTs using ion irradiation in electrolyte plasma [152,153]. Lulevich *et al.* pulled a DNA molecule out of a SWNT using an AFM probe. Since the measured force was nearly constant, they suggested that the removal of the DNA molecule was frictionless [126].

Many new small companies that commercialize the functionalization of CNT products have been established. These companies are quite valuable for physicists and biologists who do not synthesize their own CNTs. Currently, since each company proposes their own protocols, qualities of some functionalized CNTs are dependent on manufacturers even though similar structural formulas are written in their catalogs. Further accumulation of the use of functionalized CNTs is necessary for the DNA nanotechnology field.

## 9. Theoretical Studies of Nucleic Acid Adsorption to CNTs

**Figure 6:**
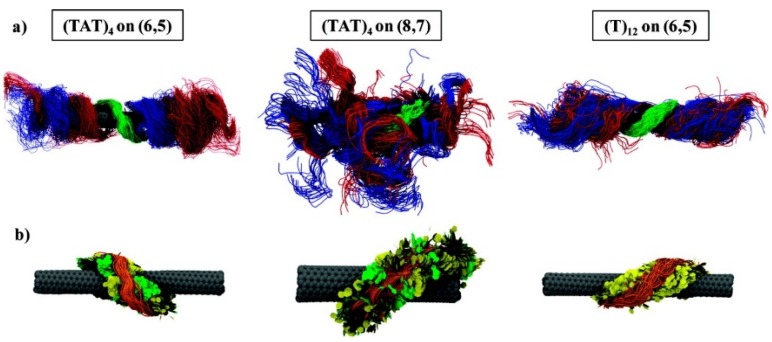
Recent theoretical models of several combinations of ssDNA and SWNTs, proposed by Roxbury *et al.* (Reprinted from reference [169] with permission).

The combination of theoretical and experimental approaches is a trend in fundamental hybridization research of nucleic acids and CNTs [155,158,159,160,161,162,163,164,165,166,167,168,169,170,171,172,173,174]. Enyashin *et al.* employed a quantum mechanical density-functional tight-binding method (DFTB) to assess the adsorption of DNA molecules onto CNT surfaces [158]. Frischknecht *et al.* performed atomistic MD simulations to measure the binding energy of six different nucleotide monophosphates (NMPs) under two distinctive environmental conditions. They established that the binding energy between the NMPs and (6,0) SWNTs is a few kilocalories/mole [159]. Roxbury *et al.* studied sequence-specific self-stitching motifs of short ssDNA using replica exchange molecular dynamics (REMD) [164]. Umadevi *et al.* demonstrated that the binding energy between CNTs and DNA/RNA nucleobases could be controlled by the curvature of the CNTs using quantum chemical calculations [165]. Xiao *et al.* studied sequence- and base-dependent interactions using all atom MD simulations and thermodynamic analyses based on their fundamental interests [166]. Akdim *et al.* examined the selectivity of DNA-CNT hybridization using density functional theory calculations [167]. While ssDNA has mainly been studied from the theoretical aspect, Alegret *et al.* investigated dsDNA-SWNT hybrids using MD simulations [168]. Their results indicated that the disruption of short dsDNA helices was accelerated in the presence of SWNTs. Roxbury *et al.* reported that DNA formed ordered structures on SWNT surfaces, and the structures are strongly dependent on the sequence of the DNA and the types of SWNTs [169]. Figure 6 depicts their theoretical models of ssDNA-SWNT hybridization with distinct sequences and chiralities. Santosh *et al.* suggested that dsDNA undergoes much less unzipping and wrapping on SWNTs within 70 ns using MD simulations [170]. Wu *et al.* indicated that bundled CNTs can be dispersed by applying torsional energy based on MD simulations [174]. Iliafar verified their experimental data using REMD [112]. Makarucha *et al.* presented a review article to summarize theoretical research in this field in 2011 [163].

In the past ten years, theoretical modeling has proven useful for examining various conditions of DNA-CNT hybridization. Theoretical models of dsDNA-CNT hybrids, not only ssDNA-CNT hybrids, have also recently been proposed. At this stage, systematic combination of theoretical and experimental studies might be required.

## 10. Biological Applications

Although much work has focused on the fundamental aspects of DNA-CNT hybridization, many recent reports have presented ideas for and demonstrations of biological applications [40,43,50,55,56,63,64,67,74,75,76,82,99,101,108,112,117,130,133,175,176,177,178,179,180,181,182,183,184,185,186,187,188,189,190,191,192,193,194,195,196,197,198,199,200]. The following describes several examples.

Biosensors comprise a widespread field in the application of DNA-CNT hybrids. Zhang *et al.* reported the selective detection of single nitric oxide (NO) molecules. In this study, (AT)_15_ ssDNA was adsorbed onto an array of near-infrared fluorescent semiconducting SWNTs, which resulted in AT_15_-SWNT hybrids [133]. Zhang *et al.* achieved label-free detection of sequence-specific DNA using MWNTs, noting that the mismatch of a single base could be detected using light scattering signals [176]. Aravind *et al.* demonstrated the detection of dopamine using ssDNA-MWNTs [180]. Cao *et al.* developed a highly sensitive immunosensor for detecting human chorionic gonadotrophin (HCG) using dsDNA-MWNT hybrids and methylene blue (MB) on a glass carbon electrode [182]. Shi *et al.* fabricated highly sensitive glucose and ATP microbiosensors using ssDNA-SWNT hybrids [185]. Thuy *et al.* detected *Escherichia coli* O157:H7 using DNA-MWNT hybrids [189]. Su *et al.* fabricated gas sensors for H_2_, H_2_S, NH_3_, and NO_2_ using metal-DNA-SWNT hybrids on microfabricated electrodes [195].

**Figure 7:**
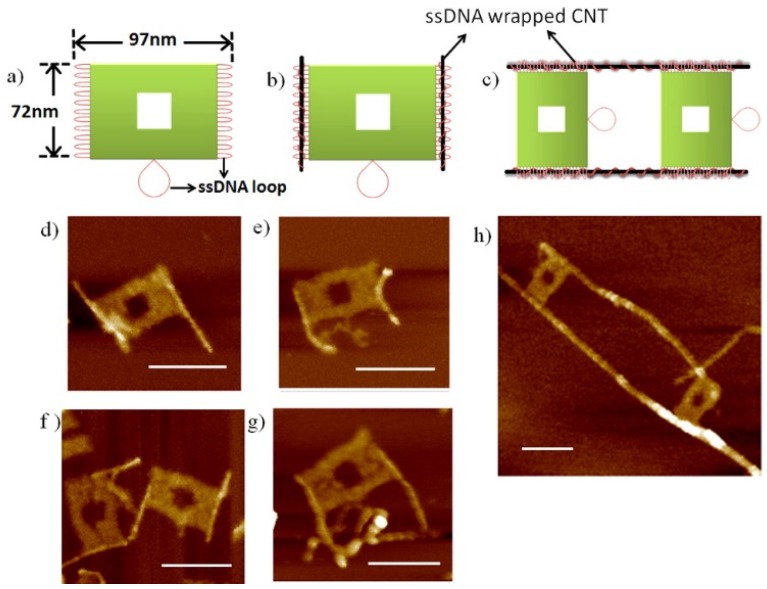
Schematic representations and AFM images of structures fabricated with ssDNA-SWNT hybrids using the DNA origami technique, generated by Mangalum *et al.* (Reprinted from reference [193] with permission).

Kawaguchi *et al.* used dsDNA-SWNT hybrids as heat delivery vehicles for the thermal ablation of tumors. Anti-human IgG was attached to the hybrids for specific binding with the targeted protein molecules [187]. The attachment of quantum dots to DNA-CNT hybrids was reported by several authors [117,190]. Gong *et al.* found that molecular structural switching of T_15_ adsorbed onto SWNTs was induced when the sample was exposed to Hg^2+^ [192]. Williams *et al.* described an inhibitory concentration of DNA-SWNTs in PCR reactions [200]. Mangalum *et al.* fabricated unique structures by combining ssDNA-SWNT hybrids and DNA origami techniques, as shown in Figure 7 [193].

Finally, the interaction between nucleic acids and graphene has becoming a major area of interest [201,202,203,204,205,206,207,208,209,210,211,212,213,214,215,216,217,218,219], and the safety of CNTs is an important area of research [220,221,222,223,224,225,226,227,228,229,230,231,232,233,234,235,236,237,238,239,240,241,242,243,244,245]. Further review articles that focus on these topics will be compiled.

The number of biological applications has increased drastically over the past several years. This suggests that biophysicists and biochemists can use DNA-CNT hybrids even though they do not specialize in CNTs. Although fundamental analysis of DNA-CNT hybridization is an important area of research, it appears that biological applications can be developed using our present understanding of DNA-CNT hybrids.

## 11. Conclusions

In this review, biological applications of CNT hybrids with nucleic acids are discussed and categorized by several key factors. This survey suggests that fundamental research of the hybridization phenomenon of nucleic acids and CNTs has continued intensively from both theoretical and experimental viewpoints. Progress in practical applications, such as fabrication of nanosensors, has recently accelerated. Some topics cannot be covered in this article, and thus, more specific review articles will be written in the future.
